# Enabling formulations of aprepitant: *in vitro* and *in vivo* comparison of nanocrystalline, amorphous and deep eutectic solvent based formulations

**DOI:** 10.1016/j.ijpx.2021.100083

**Published:** 2021-06-05

**Authors:** Henrik Palmelund, Jonas B. Eriksen, Annette Bauer-Brandl, Jukka Rantanen, Korbinian Löbmann

**Affiliations:** a University of Copenhagen, Department of Pharmacy, Universitetsparken 2, 2100 Copenhagen, Denmark; b University of Southern Denmark, Department of Physics, Chemistry and Pharmacy Campusvej 55, 5230 Odense, Denmark

**Keywords:** Bioavailability, Poorly soluble drugs, Deep eutectic solvents, Supersaturation, Amorphous, Nanocrystalline

## Abstract

A deep eutectic solvent (DES) is a eutectic system consisting of hydrogen bond donor and acceptor has been suggested as a promising formulation strategy for poorly soluble drugs. A DES consisting of choline chloride and levulinic acid in a 1:2  molar ratio was used to formulate a liquid solution of the model drug aprepitant. This formulation was tested *in vitro* (drug release and permeability) and *in vivo* (rat model) and compared with the performance of amorphous aprepitant and the commercial aprepitant nanocrystalline formulation. In this study a DES formulation is compared for the first time directly to other established enabling formulations. The *in vitro* drug release study demonstrated that the DES formulation and the amorphous form both were able to induce an apparent supersaturation followed by subsequent drug precipitation. To mitigate the risk of precipitation, HPMC was predissolved in the dissolution medium, which successfully reduced the degree of precipitation. In line with the results from the release study, an *in vitro* permeation study showed superior permeation of the drug from the DES formulation and from the amorphous form compared to the nanocrystalline formulation. However, the promising *in vitro* findings could not be directly translated into an increased *in vivo* performance in rats compared to the nanocrystalline formulation. Whilst the DES formulation (34 ± 4%) showed a higher oral bioavailability compared to amorphous aprepitant (20 ± 4%), it was on par with the oral bioavailability obtained from the nanocrystalline formulation (36 ± 2%).

## Graphical Abstract

**Figure:**
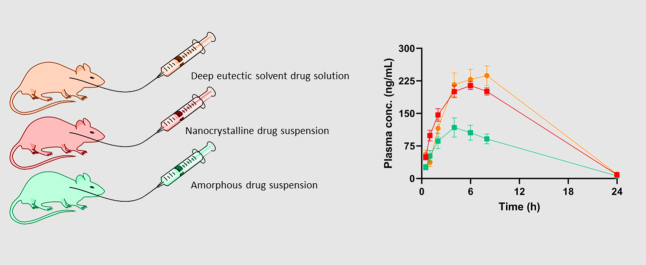

## 1 Introduction

A deep eutectic solvent (DES) is a combination of two or more components consisting of a hydrogen bond donor and acceptor. The two DES-components interact through a hydrogen bond, which causes a large melting point depression (Crespo et al., 2018). DESs have found applications in various branches of the chemical industry as non-volatile, non-flammable, and environmental friendly alternatives to organic solvents (Smith et al., 2014; Zhang et al., 2012). DESs have also been proposed as promising solvents or carriers for drugs (Morrison et al., 2009; Lu et al., 2016; Palmelund et al., 2019). It should be noted that there are many different types of DESs, depending on the nature of the components comprising the DES. In this study, for simplicity reasons, a DES is referred to a type 3 DES, *i.e.* comprising a hydrogen bond donor and acceptor. However, for a detailed a review on the complex nature of DESs, the interested reader is referred to Hansen et al. (2021), Martins et al. (2019), and Smith et al. (2014). With the increasing number of poorly soluble drug candidates, DESs possess the potential to become a part of the tool box of enabling strategies for successful formulation development of novel drugs (Morrison et al., 2009; Lu et al., 2016; Li and Lee, 2016). Here, DESs may give formulation scientists the possibility to design tailor-made DESs for a given drug candidate by selection of the DES-components and their composition (Dai et al., 2015; Palmelund et al., 2020). For example, the solubility of itraconazole has been reported to be 22,000–53,000 times higher in a DES comprising choline chloride and carboxylic acids than in water (Morrison et al., 2009; Li and Lee, 2016). The high drug solubilities in DESs are beneficial as the drug can be administered in a solution. Nevertheless, a solution with a high drug concentration eventually will lead to an apparent drug supersaturation upon dilution in the gastro-intestinal fluids and hence, carries the accompanied risk for drug precipitation. In this context, drug formulations based on DESs can be classified as a supersaturating drug delivery system (Bevernage et al., 2013).

A supersaturating drug delivery system is a drug formulation that upon oral administration generates an apparent drug supersaturation in the gastro-intestinal tract, potentially resulting in an increased flux across the gastro-intestinal barrier. Besides DESs, more established supersaturating drug delivery systems are amorphous solid dispersions or lipid based drug delivery systems (Bevernage et al., 2013). Generally, the supersaturated state is thermodynamically unstable and a supersaturated drug will eventually precipitate to obtain a thermodynamically stable system. Due to the presence of micelles in FaSSIF the thermodynamic activity is difficult to determine because the drug will partition into micelles and other colloidal species present in FaSSIF. Therefore, drug concentrations above the equilibrium solubility are referred to as apparent supersaturations (Blaabjerg et al., 2018a). The induction time for precipitation depends on the degree of apparent supersaturation according to classical nucleation theory (Nielsen, 1964; Ozaki et al., 2012). The degree of apparent supersaturation is calculated as the concentration of the supersaturated solution divided by the equilibrium solubility of the thermodynamically most stable crystalline form of the drug. If the drug precipitates, the free drug concentration will be reduced and subsequently decrease the driving force for permeation across the gastro-intestinal barrier. Thus, it is of interest to mitigate the risk of precipitation from supersaturating drug delivery systems. Polymeric precipitation inhibitors, such as HPMC, can be used to delay the nucleation and/or decrease the crystal growth rate (Guzmán et al., 2007; Bevernage et al., 2011).

We have previously found that aprepitant has a higher solubility in a DES containing choline chloride, lactic acid, and water compared with three conventional pharmaceutical solvents (ethanol, glycerol, and poly ethylene glycol 300) (Palmelund et al., 2019). Aprepitant is also able to supersaturate (Palmelund et al., 2016a), which makes it a suitable model drug for a DES-based supersaturating drug delivery system. In a typical chemotherapy, 125 mg aprepitant is given on the first day of treatment followed by 80 mg on day two and three to treat nausea and vomiting associated with highly and moderately emetogenic cancer chemotherapy. During development, a strong food effects have been reported for a tablet formulation containing the (macro-)crystalline drug (Wu et al., 2004). The food effects could be eliminated by formulation efforts in the final drug product, EMEND®, which is formulated as encapsulated beads coated with nanocrystals of aprepitant with a diameter of 200 to 300 nm (Roos et al., 2018a; Hargreaves et al., 2011). Several mechanistic pharmacokinetic studies have classified aprepitant as a biopharmaceutical classification system (BCS) class II drug, with solubility-limited oral drug absorption (Wu et al., 2004; Litou et al., 2019; Shono et al., 2010). It has been suggested that the increased absorption of aprepitant from the nanocrystalline formulation is caused by increased diffusion of dissolved drug, colloid structures and nanoparticles in the aqueous boundary layer (Roos et al., 2018a; Roos et al., 2017; Roos et al., 2018b).

Since the quest for pharmaceutical applications of DESs has only recently been embarked, the drug release from DESs has so far not yet been compared with the drug release from other enabling techniques. To our knowledge, this study is the first of its kind and investigates DES formulations towards their *in vitro* release, permeability and *in vivo* performance against two alternative enabling formulation approaches, namely the amorphous form and the marketed nanocrystalline formulations. In particular, the release and precipitation behavior of a DES formulation and the amorphous form are investigated with or without the presence of the precipitation inhibitor, HPMC. Furthermore, the *in vitro* permeability of aprepitant released from the DES formulation and the amorphous form is measured using a high throughput permeation platform that previously has shown to be able to distinguish formulations of poorly soluble drugs according to bioavailability (Jacobsen et al., 2019). Their permeations are compared against the nanocrystalline marketed formulation. Lastly, the *in vivo* performance of all three formulation approaches are compared using a rat model. Sprague Dawley male rats were dosed by oral gavage and the oral bioavailability determine by analysis of the plasma concentration.

## 2 Materials and methods

### 2.1 Materials

Aprepitant was kindly donated by Merck Sharpe & Dohme (Kenilworth, NJ, USA). All other chemicals were purchased from the respective vendors: hydroxypropyl methylcellulose (150-400 mPa·s, 2% in water at 20 °C) (HPMC; Tokyo Chemical industry, Tokyo, Japan), FaSSIF V.1 powder (biorelevant.com Ltd., London, United Kingdom), levulinic acid and choline chloride (Sigma Aldrich, Steinheim, Germany). PermeaPlain Plate was kindly donated as a gift from InnoME GmbH (Espelkamp, Germany). EMEND® 80 mg capsules were purchased from a local distributor and the beads gently mortared before use.

### 2.2 Methods

#### 2.2.1 Preparation of DES and DES formulations

The DES formulations consisting of choline chloride and levulinic acid in a 1:2  molar mixture was heated (75 °C) under magnetic stirring on a hotplate until a clear solution was formed. For the *in vitro* release and permeation studies, a DES-drug formulation containing aprepitant at 3.5 mg/g was prepared. For the *in vivo* studies, a DES-drug formulation containing aprepitant at 5.0 mg/g was prepared as a solution or as a suspension containing 10 wt% HPMC in the DES-drug solution containing 5.0 mg/g aprepitant. DES formulations were stored with silica gel in sealed glass vials.

#### 2.2.2 Solubility determination

A volume of 2 mL neat DES was magnetically stirred with an excess amount of aprepitant for 24 h at room temperature. Separation of solution and excess solid aprepitant was performed by 2✕30 min centrifugation of 1000 μL at 12,400 x G followed by centrifugation of 500 μL of the supernatant from the first centrifugation step at 12,400 x G. A quantity of the supernatant from the second centrifugation step was diluted in acetonitrile and quantified by HPLC. The solubility was determined in triplicates.

#### 2.2.3 Chemical Stability

The concentration of aprepitant in DES (3.5 mg/g) was quantified at the time of preparation and at week 1 and 2 after preparation and storage in a closed vial at 40 °C. The chemical stability was determined in triplicate and the recovered concentration was statistically evaluated using a one-way ANOVA test in GraphPad Prism version 8.4.2 (San Diego, CA). The recovery was performed in triplicates.

#### 2.2.4 Preparation of amorphous aprepitant

Aprepitant has previously been reported to be a good glass former belonging to the GFA class 3R (Blaabjerg et al., 2018b). In the present study, aprepitant was placed on aluminium foil and heated on a hotplate set to 250 °C. The aluminium foil was removed from the hotplate immediately after the material was fully molten. The molten aprepitant solidified within 30 s to an amorphous glass that was gently powdered using a mortar and pestle, and subsequently analyzed by X-ray powder diffractometry (XRPD, see Section 2.2.6). Amorphous material was stored with silica gel in sealed glass vials.

#### 2.2.5 Drug release

The drug formulations were weighed into cylindrical glass vials before 20 mL of FaSSIF dissolution medium was added with or without 0.05 *w*/*v*% predissolved HPMC. The dissolution medium was magnetically stirred by a cross magnet at 100 rpm while the temperature was maintained at 37 °C using the experimental setup from the μDISS Profiler™ (Pion, Billerica, MA). Samples (1 mL) were withdrawn and replaced after 5, 10, 15, 20, 30 and 60 min. The samples were centrifuged at 12,400 x G for 1 min. Subsequently, 200 μL of the supernatant was then diluted 1:4 (*v*/v) with acetonitrile to precipitate the phospholipids and centrifuged for an additional 1 min before 500 μL of the supernatant was collected for quantification. The accumulated amount of released aprepitant was calculated considering the volume of replaced dissolution medium. All drug release experiments were performed in triplicate.

#### 2.2.6 Solid state characterization

After 60 min of drug release from the DES formulations (4 mg dose) and the amorphous form (3 and 40 mg dose), the undissolved drug and/or precipitates were collected by centrifugation 4696 x G for 5 min of the dissolution medium. The pellets were collected and analyzed by XRPD using a PANalytical X'Pert PRO X-ray diffractometer equipped with a PIXcel detector (Almelo, The Netherlands) using Cu Kα radiation (1.54187 Å), at 45 kV and 40 mA. The pellets were analyzed from 5 to 30°2θ/using a step size of 0.02626°2θ with a speed of 0.06734°2θ/s. The data were collected using X'Pert Data Collector and analyzed using X'Pert HighScore (PANalytical B.V., Almelo, The Netherlands). The reference diffraction patterns for crystalline aprepitant form I and II were accessed *via* Cambridge Structural Database and XRPD patterns were calculated using the Mercury software version 4.1.3 from Cambridge Structural Database, CSD (Cambridge, UK), CSD references GOPDUK01 (form I) and GOPDUK02 (form II) (Braun et al., 2008). The pellets were also analyzed by polarized light microscopy using a Carl Zeiss AG 47 50 57 stereo microscope (Oberkochen, Germany) equipped with an Evolution MP camera from Media Cybernetics (Rockville, MD, USA) controlled by the Q Capture software from Q Imaging (Surrey, BC, Canada). The pellets were placed underneath a cover slide and examined using a DMLM microscope from Leica Microsystems GmbH (Wetzlar, Germany), operated in polarized light microscopy (PLM) mode using a 20× magnifying objective.

#### 2.2.7 Permeation setup

The PermeaPlain Plate from InnoMe (Espelkamp, Germany) was used to study the drug release/permeation interplay from different formulations of aprepitant. The 96-well plate setup consisted of an acceptor plate with a permeable cellulose-based membrane in the bottom placed on top of a donor plate and sealed with adhesive sealing foil (x-Pierce., Excel Scientific, Inc.). In the donor compartment, 300 μL of FaSSIF with or without 0.05 *w*/*v*% predissolved HPMC was added. The formulations were tested by dispersing a quantity of the formulations corresponding to 150 μg/mL aprepitant in the donor media right before the experiments started. The nanocrystalline formulation was only tested in FaSSIF. In the acceptor plate, 120 μL 2 *w*/*v*% D-α-Tocopherol polyethylene glycol 1000 succinate solution was added to maintain sink conditions in the acceptor compartment throughout the experiment. The plate was incubated in a Thermo-shaker PHMP-4 (Grant-bio; England) at 37 °C and shaken at 300 rpm. At each sampling time, 80 μL was withdrawn from acceptor wells and directly quantified by HPLC, with no acceptor wells reused for more time points. The experiments were performed as quadruplicate (*n* = 4). Before the experiments, adhesion of aprepitant to the well plates was tested. The statistical differences between the formulations were evaluated using a two-way-ANOVA test in GraphPad Prism version 8.4.2 (San Diego, CA).

#### 2.2.8 Aprepitant quantification

Aprepitant was quantified by high performance liquid chromatography (HPLC). In the drug release and stability experiments, aprepitant was quantified by an Agilent 1260 infinity LC system with an Aglient 1290 infinity pump and DAD detector from Agilent Technologies (Glostrup, Denmark). All samples were injected on a Kinetex®, 5 μm EVO C18, 100 × 4.6 mm column from Phenomenex (Værløse, Denmark). A flow rate of 1 mL/min was applied with a mobile phase consisting of 55 *v*/v% acetonitrile and 45 v/v% 25 mM ammonium acetate. For the permeation study, aprepitant was quantified by a Waters 2695 HPLC system with a Waters 2487 DAD detector. All samples were injected on the column described above and with the same injection volume. The mobile phase composition was changed to 45 *v*/v % acetonitrile and 55 v/v % 25 mM ammonium acetate in order to separate the aprepitant peak from additional peaks originating from the glue used to attach the membrane to the acceptor plate of the PermeaPlain Plate. The method was validated using recovery test and sensitivity analysis for pH and mobile phase composition. Standard solutions were analyzed before and after the analysis of samples. A single standard was also analyzed every hour to confirm the validity of the results.

#### 2.2.9 Pharmacokinetic study

*In vivo* studies were performed with 23 Sprague Dawley male rats with an average weight of 325 g at the time of the experiments. The rats were brought to the animal facility 5 days before the experiments to ensure a proper acclimatization. The animal health and welfare were monitored and recorded before and during the experiments. The animals were allowed access to food and water *ad libitum* during the acclimatization and experiments. The pharmacokinetic study was designed with five arms as seen in.

Table 1. One group (3 animals) receiving 2 mg/kg body weight (bw) intravenously and the other four groups (5 animals each) receiving the aprepitant dose by oral gavage. The intravenously solution had an aprepitant concentration of 2 mg/mL dissolved in PEG400, ethanol and water in a 60:20:20 volume ratio. Two groups of five animals each were given suspensions of either the commercial product EMEND or amorphous aprepitant at 2.4 mg/kg bw. The suspensions were administered in a 0.5 *w*/*v*% HPMC solution with a dosing volume of 5 mL/kg bw. An additional two groups of five animals each were given a 2.4 mg/kg bw dose of aprepitant dissolved in DES (5.0 mg/g) with or without 10 wt% of HPMC. The two DES formulations (with or without HPMC) were administered by oral gavage without any dilution. The oral dose of the DES formulation was based on the maximum tolerated single dose of levulinic acid in rats (Bergfeld et al., 2020) and the maximum drug loading (5.0 mg/g) that could be achieved without reaching the solubility limit. Blood samples of approximately 200 μL were drawn from *v. sublingualis* after 0.5, 1, 2, 3, 4, 6, 8, and 24 h. All animal experiments were performed in compliance with the Danish laws regulating experiments on animals and EC Directive 2010/63/EU (license no. 2016-15-0201-01094) approved by the Danish Animal Experiments Inspectorate, The Danish Veterinary and Food Administration, Ministry of Environment and Food.

**Table 1:** Pharmacokinetic study design.

Formulation	Average body weight (g)	Animals (n)	Dose (mg/kg bw)	Dose volume (mL/kg)	Dose volume (mL)
IV	340 ± 2	3	2.00	1.20	0.41
Nanocrystals	344 ± 7	5	2.40	5.00	1.72
Amorphous form	343 ± 7	5	2.40	5.00	1.72
DES formulation	350 ± 10	5	2.40	0.43	0.14
DES formulation with 10 wt% HPMC	357 ± 11	5	2.40	0.47	0.16

#### 2.2.10 Bioanalysis

Blood samples of 200 μL were collected and stabilized by K_3_EDTA in 0.5 mL Sarstedt tubes. Prior to centrifugation, the samples were stored on ice for a maximum of 20 min. Samples were centrifuged for 10 min at 1270 x G at 4 °C, and the plasma was kept below −70 °C until further analysis. The plasma samples were analyzed against imipramine as an internal standard on a Thermo Vanquish UPLC system connected to a Thermo TSQ Altis. Mobile phase A comprised MilliQ water with 0.1% formic acid and mobile phase B comprised acetonitrile with 0.1% formic acid. A gradient with 95% at 0 min, 5% at 0.9 min and, 0% of mobile phase A at 1.35 min, was applied with a flow rate of 0.8 mL/min. The samples were injected on a Phenomenex Kinetex Biphenyl C18 100A, 2.6 μm, 2.1 × 500 mm column held at 65 °C. The mass spectrometer was used with positive electrospray ionization. The MS source used 3500 V for positive ion spray with a vaporizer temperature of 350 °C and 380 °C for the ion transfer type. The sheath gas and aux gas were 60 and 15 Arb, respectively. The fragmentation transitions for multiple reaction monitoring were *m*/*z* 535.15 → 179.17 and 277.13 for aprepitant and m/z 218.15 → 86.13, 58.07, and 193.13 for imipramine.

#### 2.2.11 Pharmacokinetic analysis

The bioavailabilities were calculated using the trapezoid method. The elimination phase could not be properly determined due to the limited sampling time points and the uncertainty of the exact *T*_*max*_ value for the DES formulations. Therefore, only the bioavailability during the first 8 h after administration was calculated. The concentration at 0 min for the intravenous dose was calculated by extrapolation of the elimination phase. The bioavailability is presented with the standard error of the mean. The statistical significance was tested using a one-way ANOVA test with a 0.05 level of significance.

## 3 Results & discussion

The solubility of aprepitant in the DES was determined at 6.78 ± 0.03 mg/g, which is 321-fold higher than in FaSSIF and 1057-fold higher than in water (Wu et al., 2004; Palmelund et al., 2016b). The chemical stability of aprepitant in the DES solution demonstrated no significant difference in recovery from the time of preparation to 2 weeks after preparation.

### 3.1 *In vitro* drug release

The *in vitro* drug release was investigated for both the DES formulations and amorphous aprepitant. It was not possible to measure the drug release from the marketed nanocrystalline formulation due to incomplete separation of the nanocrystals after filtration with either a 0.1 μm pore size-filter or centrifugation.

**Fig. 1:**
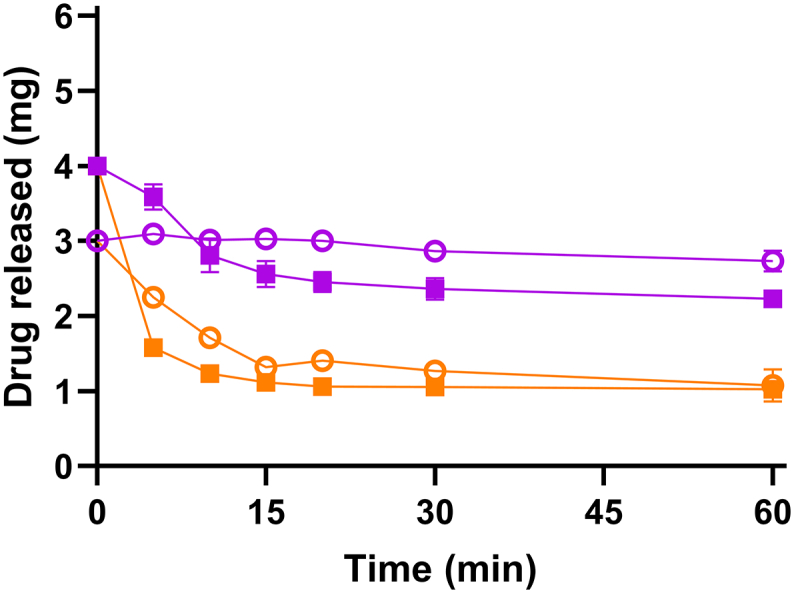
Release of 3 mg (○) and 4 mg (■) aprepitant formulated in the DES introduced to 20 mL FaSSIF (orange) or with FaSSIF with 0.05 *w*/*v*% HPMC (purple). Error bars represent standard deviations, *n* = 3. (For interpretation of the references to colour in this figure legend, the reader is referred to the web version of this article.)

Aprepitant was introduced to FaSSIF medium from a DES formulation containing 3.5 mg/g aprepitant. The formulation was added at two different target concentration levels to obtain a final drug amount of 3 or 4 mg aprepitant in 20 mL release medium. Initially, a clear supersaturated solution was obtained and subsequently precipitation of aprepitant occurred as seen by the sudden drop in the amount of released aprepitant (Fig. 1). For the 3 and 4 mg drug dose, the apparent supersaturation was maintained for 4 and 2 min, respectively, before precipitation was visually observed. In the presence of 0.05 *w*/*v*% HPMC, the precipitation onset was delayed and its extent decreased for the 4 mg dose and precipitation was prevented for the 3 mg dose (see Fig. 1).

**Fig. 2:**
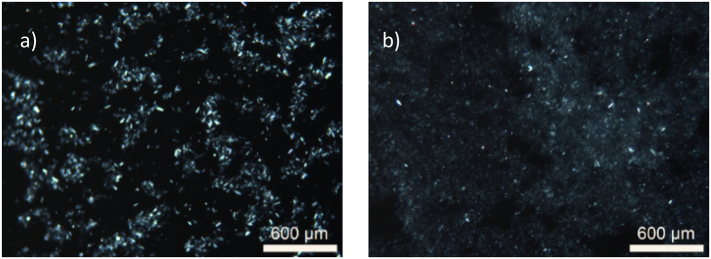
Typical polarized light microscopy images of the precipitates of 4 mg aprepitant introduced by a DES formulation in 20 mL (a) FaSSIF and (b) FaSSIF with 0.05 w/v% HPMC.

**Fig. 3:**
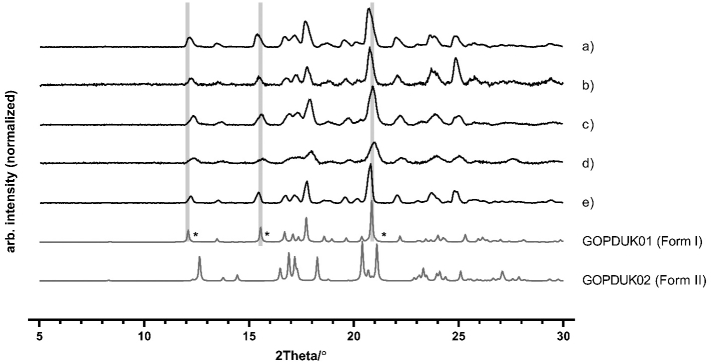
Powder X-ray diffractograms of aprepitant form I and II and the precipitates developed by DES-induced apparent supersaturation (4 mg) in 20 mL (a) FaSSIF or in (b) FaSSIF with predissolved HPMC. In addition, the diffractograms for the suspended solid material, resulting from undissolved starting material and/or precipitated drug during the dissolution of 40 mg amorphous aprepitant in 20 mL (c) FaSSIF or in (d) FaSSIF with predissolved HPMC. The diffractogram shown in (e) is the bulk material used for the DES formulations and preparation of amorphous aprepitant. The star symbol (*) represents the assigned crystal form for the most characteristic diffraction peaks. The reference diffractograms of form I and II were calculated from (Braun et al., 2008) using Mercury software.

The precipitates for each sample were collected after 60 min and analyzed by polarized light microscopy and XRPD. The polarized light microscopy indicated birefringence that is typical for crystalline matter, as well as a notable difference in particle size between the two crystalline precipitates from the 4 mg dose (Fig. 2). The precipitate from FaSSIF in the presence of HPMC showed overall a smaller particles compared with the precipitate collected from FaSSIF. The crystalline nature of the precipitates from both media were identified as form I, as indicated by the XRPD diffractograms (Fig. 3). Form I has been reported to be the thermodynamically most stable form of aprepitant (Braun et al., 2008). The pH in both media was determined at 3.40 ± 0.05 after 60 min, which is lower than the pH of FaSSIF (pH = 6.50), which can be attributed to the presence of the DES components. When an apparent supersaturation was introduced by a dimethyl sulfoxide solution of aprepitant, similar precipitation kinetics were observed in FaSSIF with or without predissolved neat DESs (data not shown). Thus, the presence of dissolved DES-components in FaSSIF and the resulting pH change did not influence the onset of precipitation.

Aprepitant is weakly basic with a pKa of 2.4 and weakly acidic with a pKa of 9.7. Hence, the molecule will become ionized at low pH, which will impact its solubility. The aqueous solubility of aprepitant has been reported to be 130 μg/mL at pH 1, but varies only between 3 and 7 μg/mL in the pH range from 2 to 7 (Olver et al., 2007). Therefore, the lowering of the pH caused by levulinic acid did not significantly influence the solubility and precipitation kinetics.

**Fig. 4:**
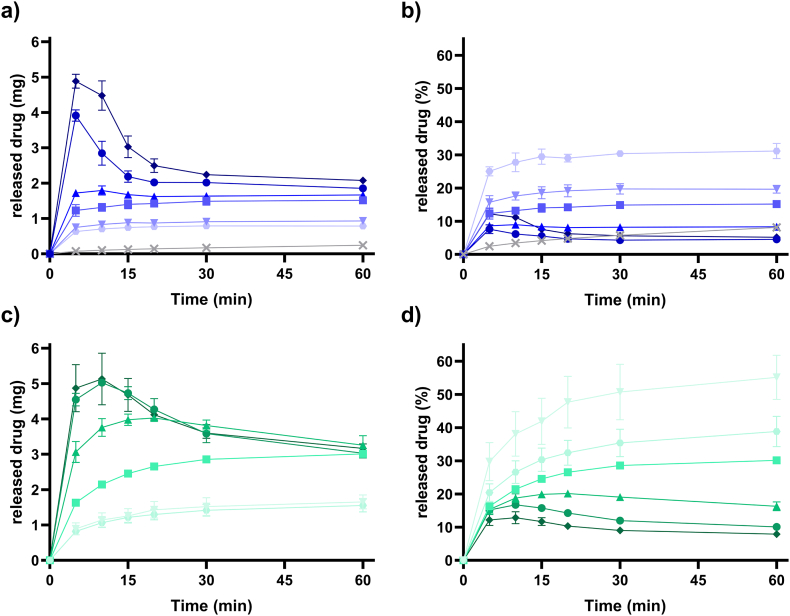
Release of amorphous aprepitant presented in amount and percentage in FaSSIF (a and b) and FaSSIF with predissolved 0.05 w/v% HPMC (c and d). The amount of amorphous aprepitant is ▼3, ⬣ 4, ■ 10, ▲20, ● 30, and ◆ 40 mg. 3 mg of crystalline aprepitant (X) in FaSSIF is presented in grey (a and b). Error bars represent standard deviations.

The release of amorphous aprepitant showed a significantly higher dissolution rate and higher solubilities than for the crystalline form I of the aprepitant bulk material (Fig. 4). Amorphous aprepitant was able to induce an apparent supersaturation and the degree of apparent supersaturation was dependent on the dose of amorphous aprepitant (drug doses at 3, 4, 10, 20, 30 and 40 mg). Different doses of amorphous aprepitant were investigated to identify the dose that would achieve an equivalent apparent drug supersaturation as induced by the 3 mg dose from the DES formulation in the presence of HPMC. For the 3, 4, and 10 mg aprepitant dose, the released drug reached a maximum amount of released drug (AR_max_) which exceeded the equilibrium solubility of aprepitant. However, precipitation was not observed (Fig. 4a). For the drug doses of 20, 30, and 40 mg of amorphous aprepitant, a higher degree of apparent supersaturation was obtained in FaSSIF, which in turn initiated precipitation from the solutions. As expected, the presence of predissolved HPMC delayed the onset of precipitation as well as the precipitation rate for these three systems. This was seen by the later onset of AR_max_ and the higher amount of released drug after 60 min (Fig. 4c). As a result of the inhibited precipitation, the dissolution of the amorphous aprepitant was facilitated and improved performance for all investigated drug doses with respect to the concentrations obtained.

The undissolved and/or precipitated material was collected after 60 min and analyzed by XRPD. The X-ray diffractograms from FaSSIF and FaSSIF with predissolved HPMC both revealed crystalline peaks identified as the crystalline form I of aprepitant (see Fig. 3).

At AR_max_ the total amount of released aprepitant varied from 7.6% to 31% in FaSSIF (Fig. 4b). In the presence of HPMC, the amount of released drug was increased to values between 12.8% and 55% at AR_max_ (Fig. 4d). The highest percentage of released amorphous aprepitant was observed for the 3 mg dose (55%) in the presence of HPMC. The lowest percentage of released amorphous aprepitant was observed for high amounts of amorphous aprepitant due to the precipitation induced by the higher apparent supersaturation and the expression as percentage.

In the presence of HPMC, an equivalent amount of dissolved aprepitant compared to the 3 mg of aprepitant from the DES formulation would roughly be achieved by the 20 mg dose of amorphous aprepitant. In the absence of HPMC, an equivalent amount of released drug would approximately be obtained by 10 mg of the amorphous form. However, at the end of the experiment (60 min), also the 3 mg amorphous aprepitant dose obtained similar concentrations as seen from the fast precipitating DES formulation (3 mg dose). Thus, the overall best performing drug dose was 3 mg for both the amorphous form and the DES formulation. For this dose, the highest drug release percentages and no precipitation were observed for both the amorphous form and DES formulation. Therefore, the 3 mg dose was further used in the *in vitro* permeation study.

For the highest dose of 40 mg amorphous aprepitant in FaSSIF, a total of 4.48 mg was dissolved after 5 min. Thus, it was expected that the lowest dose of 3 mg would completely dissolve during the experiment. However, only 30% of the 3 mg dose was dissolved. XRPD analysis of the undissolved material from the 3 mg dose after 60 min showed the presence of crystalline material, which explains why the lowest dose of 3 mg did not fully dissolve. Thus, recrystallization and dissolution occurred simultaneously resulting in an incomplete dissolution regardless of the dose. The achieved degree of apparent supersaturation at AR_max_ was dependent on the dose, because the higher dose largely increases the dissolution rate and thus the degree of apparent supersaturation before recrystallization. A similar dissolution behavior of amorphous drugs has previously been reported (Plum et al., 2020). When the DES formulation was introduced to FaSSIF, apparent supersaturation was induced before precipitation occurred. The presence of HPMC inhibited the extent of aprepitant precipitation from the apparent supersaturation induced by either the DES formulation or amorphous aprepitant. Since aprepitant was in a dissolved state in the DES formulation, a lower dose of aprepitant was needed to induce an apparent supersaturation. This suggested that a lower drug dose in a DES formulation potentially could result in a similar bioavailability than a higher dose of amorphous aprepitant.

### 3.2 *In vitro* permeation

**Fig. 5:**
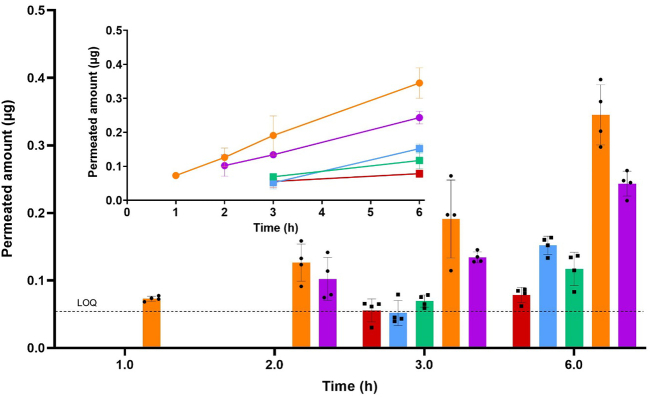
In vitro drug permeation from the nanocrystalline formulation (red), amorphous aprepitant (blue), amorphous aprepitant in the presence of HPMC (green), DES formulation (orange), DES formulation in the presence of HPMC (purple). All formulations were tested with a concentration of 150 μg/mL in the donor compartment corresponding to the 3 mg dose in the drug release studies. Error bars represent standard deviations. n = 4. Data points below the LOQ have been excluded. (For interpretation of the references to colour in this figure legend, the reader is referred to the web version of this article.)

In order to make a comparison of the DES formulation and amorphous aprepitant to the marketed nanocrystalline formulation, an *in vitro* combined release/permeation study was performed using a plain diffusion membrane. The analysis of the permeated amount of drug in the acceptor compartment allowed a comparative assessment of the nanocrystalline formulation since the undissolved nanocrystals in the donor chamber were not able to permeate the membrane. The permeation of aprepitant from the different formulations across a PermeaPlain membrane is presented in Fig. 5. The concentrations of aprepitant in all donor compartments were set to a target concentration of 150 μg/mL, corresponding to the best performing drug dose (3 mg) in the drug release experiments. The permeated amount of aprepitant was lowest from the nanocrystals. For amorphous aprepitant, a slightly higher amount of permeated drug was measured after 6 h and there was no significant difference in the concentrations obtained in the presence or absence of HPMC. Hence, the higher concentrations obtained for amorphous aprepitant in the presence of HPMC observed in the release study did not translate into an increase in permeation. The highest permeation of aprepitant was measured from the DES formulations and the amount of permeated drug after 6 h was 1.4-fold higher in pure FaSSIF than in FaSSIF with pre-dissolved HPMC. Compared to the nanocrystalline and amorphous formulation, the amount of permeated drug from the DES formulation after 6 h in FaSSIF was 4.4- and 2.3-fold higher, respectively. Data points below the LOQ have been excluded from Fig. 5.

The low permeability for the nanocrystalline formulation was most likely due to the slower dissolution and lower concentrations obtained from the crystalline drug. As seen in Fig. 4a and b, the drug release from the crystalline bulk material was much lower than for the amorphous form of aprepitant. However, the nanocrystalline drug from EMEND® can be expected to dissolve faster than the crystalline bulk material used in Fig. 4. Nevertheless, the permeated amount of drug from the nanocrystals remained lower than for the other formulations. The *in vitro* permeation was either unchanged or decreased in the presence of HPMC. A decreased drug permeation/absorption from an apparent supersaturated state in the presence of HPMC has also previously been reported *in vitro* for loviride (Bevernage et al., 2012) and *in vivo* for tadalafil and indomethacin (Strindberg et al., 2020). Absorption occurs by passive diffusion, and the presence of polymers such as HPMC may decrease the diffusion coefficient of the drug due to steric hindrance and/or increased viscosity of the gastro-intestinal fluid (Dahan et al., 2016). Thus, despite the inhibitory effect of HPMC on the precipitation, the overall drug absorption was decreased in the presence of HPMC (Bevernage et al., 2012).

### 3.3 *In vivo* oral bioavailability

**Fig. 6:**
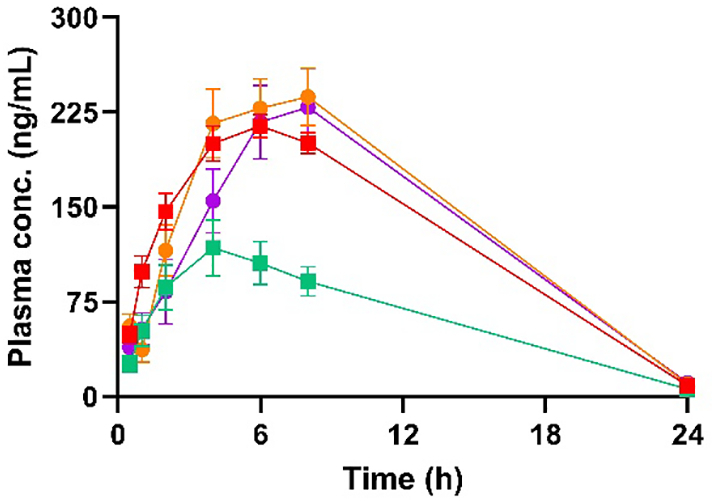
Plasma concentration in rats after oral administration 2.4 mg/kg bw of aprepitant in a nanocrystalline formulation (red), amorphous aprepitant (green) and DES formulation with (purple) and without 10 wt% HPMC (orange). Error bars represent the standard error of the mean. (For interpretation of the references to colour in this figure legend, the reader is referred to the web version of this article.)

For the *in vivo* study, the oral bioavailability was determined for the nanocrystalline marketed drug formulation, amorphous aprepritant and two DES formulations. The nanocrystals and amorphous powder were suspended in 0.5 *w*/*v*% HPMC solution to ensure a homogenous suspension for dosing. The DES formulation was studied with or without 10 wt% of HPMC suspended in the liquid DES formulation. As liquid formulations of DES potentially will be dosed in capsules, the amount of HPMC added to the DES formulation was based on the weight of the DES formulation filled into a size 0 capsule. The *in vivo* data in Fig. 6 show the plasma concentrations of aprepitant after oral administration of the four formulations. The pharmacokinetics parameters for the different formulations are summarized in Table 2. The bioavailabilities (0-8 h) were 36 ± 2% and 20 ± 4% for the nanocrystalline and amorphous formulations, respectively. The bioavailabilities (0-8 h) of the DES formulation containing HPMC showed slower absorption kinetics and a lower bioavailability (41 ± 6%) compared with the DES formulation without HPMC (34 ± 4%). The bioavailability of the DES formulation without HPMC was comparable to the nanocrystalline formulation and approximately two-fold higher than that for amorphous aprepitant.

**Table 2:** Pharmacokinetic parameters for the four different formulations of aprepitant.

Formulation	C_max_ (ng/mL ± SD)	T_max_ (h)	AUC_0-8h_ (ng∙h/mL) ± SD)	F (% ± SEM)
IV	–	–	2927 ± 565	100
Nanocrystals	214 ± 18	6	1257 ± 192	36 ± 2
Amorphous form	118 ± 44	4	690 ± 286	20 ± 4
DES formulation	237 ± 45	8	1198 ± 317	34 ± 4
DES formulation with 10 wt% HPMC	229 ± 62	8	991 ± 321	28 ± 4

*n* = 5 animals per group with the exception of the IV group with 2 animals (one rat showed subcutaneous absorption behavior).

The absorption kinetics were slowest from amorphous aprepitant, and at T_max_ (4 h) the absorbed amount of aprepitant was lower than for the other formulations after 4 h. The absorption kinetics of the nanocrystalline formulation were faster than for the other formulations. Consequently, the T_max_ for the nanocrystalline formulation at 6 h was reached earlier than for the two DES formulations. The T_max_s for the DES formulations could not properly be determined because the maximum plasma concentration was measured at 8 h with the next sampling point at 24 h.

The statistical analysis showed that the nanocrystalline formulation and the two DES formulations were significantly different from the amorphous formulation. However, no significant difference was found between the two-DES formulations and the nanocrystalline formulation. It is likely that the T_max_ for the DES formulations occurs >8 h. The slower absorption kinetics for the DES formulations compared with the nanocrystalline formulation suggests that the diffusion of dissolved aprepitant from the intestinal lumen to the intestinal barrier was more restricted than for the nanoparticles. Precipitation of aprepitant is another possibility that can explain the slower absorption kinetics. This hypothesis cannot be confirmed without aspiration of gastro-intestinal fluid after administration (Hens et al., 2016). No toxic symptoms of the DES were observed during the study.

Despite a low *in vitro* permeability shown by the dialysis-type transport mechanism, the *in vivo* bioavailability (0–8 h) of aprepitant from the nanocrystalline formulation was significantly higher than for the amorphous aprepitant and on par with the DES formulation without HPMC. This suggests that the *in vivo* absorption mechanism of aprepitant from the nanocrystalline formulation is different than from the amorphous and DES formulations. A previous study of the absorption mechanism of aprepitant has suggested that the nanocrystals are able to reduce the resistance of the aqueous boundary layer next to the intestinal barrier (Roos et al., 2017). Another study proposed that particle diffusion through the mucus layer increases the absorption of aprepitant (Roos et al., 2018b). Particle diffusion through the mucus layer has also been reported to increase the bioavailability of other drugs (Guo et al., 2019).

Previous attempts to prepare enabling formulations of aprepitant include an amorphous solid dispersion with a drug loading of 14.3 wt% in Soluplus®, in which the relative bioavailability was 93.1% of the commercial nanocrystalline formulation (Liu et al., 2015). Soluplus® increased the dissolution rate and might also have inhibited precipitation and hence improved the bioavailability as seen for pure amorphous aprepitant in this study.

## 4 Conclusion

This study investigated the *in vitro* and *in vivo* performance of three enabling formulations of the poorly soluble drug aprepitant. The *in vitro* drug release demonstrated that it was possible to obtain a higher percentage of released aprepitant from a DES formulation compared to amorphous aprepitant. In addition, apparent supersaturation was observed for both formulation approaches, which could be maintained for a prolonged period of time in the presence of HPMC. The *in vitro* combined release/permeation study showed that although HPMC inhibited precipitation, the permeation was decreased in the presence of HPMC. In line with the results from the *in vitro* release study, the *in vitro* permeation showed the largest fraction of permeated drug for the DES formulations followed by the amorphous and nanocrystalline formulations. These results demonstrate that it was possible to design DES-based supersaturating systems. However, the promising *in vitro* findings could not be translated into an increased *in vivo* performance compared to the nanocrystalline formulation. The *in vivo* study revealed an equivalent bioavailability of the DES (34 ± 4%) and nanocrystalline formulation (36 ± 3%). The bioavailability of amorphous aprepitant was significantly lower (20 ± 4%). Consequently, while DES based formulations proved to be a promising enabling formulation strategy *in vitro*, more insight into their *in vivo* performance is necessary to potentially allow to translate the findings into better formulations and thereby, benefit from their good *in vitro* behavior also *in vivo*.

## Declaration of Competing Interest

The authors have no conflict of interest to declare.
